# Crowdsourced Mapping for Healthy Food Accessibility in Dallas, Texas: A Feasibility Study

**DOI:** 10.3389/fpubh.2020.00071

**Published:** 2020-03-06

**Authors:** Thomas McKey, Dohyeong Kim, SungChul Seo

**Affiliations:** ^1^School of Economic, Political and Policy Sciences, University of Texas at Dallas, Richardson, TX, United States; ^2^Department of Environmental Health and Safety, College of Health Industry, Eulji University, Seongnam, South Korea

**Keywords:** food deserts, crowdsourcing, geographic information, accessibility, grocery stores

## Abstract

Since its first use for describing a neighborhood lacking access to food in the 1990's, “food deserts” has been widely addressed by researchers and adopted as an indicator of neighborhood-level food insecurity by governmental agencies, such as USDA. However, mostly due to cost and difficulty in collecting georeferenced data and characteristics of grocery stores, the USDA Food Access Research Atlas is infrequently released, and considers only income, vehicle ownership, and distance to the nearest grocery store. In this paper, we explored the feasibility of a crowdsourced geospatial data source, coupled with additional measures, in supplementing the USDA's current designation of food deserts. We used Yelp data to map food deserts in the city of Dallas and compared them with those based on the 2015 USDA food retailer database. Although direct comparison was not possible due to time mismatch between the two data sources, the discrepancies highlighted the need of a more frequent identification of food deserts for timely policy intervention. Furthermore, we extended mapping to reveal other potential areas of concerns, by adding the Transit Score metric and Yelp's price descriptor of businesses. The resulting maps illustrated the areas with grocery stores nearby but with limited accessibility due to lack of public transit or potential financial barriers in purchasing foods due to high prices. Our findings demonstrate the current status and future potential of up-to-date crowdsourced, georeferenced data as a complement of official government data, which could serve to extend food access research and guide health policies.

## Introduction

For almost two decades, the food environment has been the focus of research covering food access disparities or more commonly labeled “food deserts” (1–3). Eating a nutritious diet is encouraged when healthy foods are convenient, which aids in preventing the development of chronic diseases (4, 5). Disparities in access to fresh and healthy foods are well-documented (3, 6–8), but several obstacles have limited the ability of national and local governments to thoroughly address these issues. One of these obstacles is related to the difficulty in defining and measuring access to nutritious food in an objective and comprehensive manner (9). The popular classification of food deserts, developed by the US Department of Agriculture (USDA) Food Access Research Atlas, provides a standard for identifying low-access to healthy food across the United States. However, the multifactorial processes of describing food access and consumer behavior call for a more comprehensive food access metric that accounts for more than just income, vehicle ownership, and distance to the nearest supermarket (10–12). A fair amount of research demonstrates factors that influence healthy food access that are not part of the USDA's definition of a food desert, and direct observations of shortages in accessible grocery stores do not align with the identified food deserts based on the USDA's definition (8, 10, 13). Several recent studies emphasized the importance of considering residents' mobility, facilitated by public transit systems, when identifying food deserts (14, 15). Other studies have shown that food price plays an important role in where people choose to shop for food (3, 16, 17). Without accounting for the price of food, defining access and identifying the population with limited access to healthy food would remain inadequate (18). However, collecting data on the prices of food items at grocery stores is a cumbersome process, which typically involves *in-situ* data collection at individual grocery store locations. Moreover, the USDA's Food Access Research Atlas is typically published every 4–5 years (as of 2019, the most recent release was 2015), which fails to incorporate the dynamic changes in the grocery retail market. According to the Jones Lang LaSalle (JLL)'s 2019 Grocery Tracker report, new grocery store openings went up 30% in 2018 and more than one-quarter of these stores were in Florida, California, and Texas (19).

Crowdsourced data have been used to supplement existing data sources by incorporating local-level detailed information, but they have not been fully employed in food access research (20). Yelp is an open source of near real-time data with shopper-provided price descriptions for businesses across the US and abroad, but few recent studies have used its data within the public health domain. A study seeking to identify the number of hookah bars in New York City used Yelp data to identify their distribution and monitor their presence over time (21), while another study built predictive models for health code violations in 440 restaurants using Yelp data (22). Another study, that classified restaurants by taste, found associations between restaurants labeled with certain tastes and nearby neighborhood characteristics, such as income, racial composition, and education (23). However, only a couple of studies have used Yelp data in healthy food access research (24, 25). These studies demonstrated that Yelp data contained more accurate details on restaurants and healthy food stores than traditional business information sources and commercially available datasets, but had coverage limitations over a metro area. Although these studies show the potential of Yelp data, there is still a need of more empirical studies that can integrate relevant information available on Yelp with other databases to evaluate its feasibility to improve and supplement the USDA's existing designation of food deserts. Results from integrating these data sources can be particularly beneficial for people living in low-income households who have grocery stores nearby but do not own a personal vehicle or are far from public transportation, since their communities lack accessibility to healthy food but are not designated as a food desert according to the current USDA designation. Similarly, low-income communities near grocery stores would still face limited accessibility to healthy food if they cannot afford to purchase this food due to high prices. No study has yet demonstrated how many communities fall into these categories and how they are spatially distributed. Fortunately, several data sources for local area factors related to healthy food access, such as transit scores and Yelp price indicators for grocery stores, have recently become available to the public.

Thus, this study evaluated the feasibility of Yelp data as a free and real-time data source for crowdsourced mapping for healthy food accessibility and discussed how the data could be used to improve the traditional healthy food accessibility classification developed by the USDA Food Access Research Atlas. In addition, using the Transit Score metric and a price descriptor available through Yelp data, this research identified neighborhood areas with access to grocery stores but with transportation issues due to a lack of public transit or potential financial barriers in purchasing foods from nearby grocery stores. The findings from this study demonstrated the current status and future potentials of Yelp data's up-to-date nature and user-provided information on businesses in extending food access research, which highlighted the roles of crowdsourced geographic information to measure community resources directly linked to determinants of health.

## Methods

### Food Deserts Mapping Using USDA Data

The USDA defines food deserts as a composite indicator of low-income and low-access. A low-income census tract is defined as “a tract with either a poverty rate of 20 percent or more, or a median family income <80 percent of the state-wide median family income; or a tract in a metropolitan area with a median family income <80 percent of the surrounding metropolitan area median family income” (26). A low-access census tract is “a tract where a significant number (at least 500 people) or share (at least 33 percent) of the population is >1.0 mile from the nearest supermarket, supercenter, or large grocery store for an urban area or >20 miles for a rural area” (26). To map food deserts based on the USDA classification applied to 2015 USDA food retailer database (most recent release at the time of this study), the combined list of supermarkets and large grocery stores that met the USDA definition (containing all the major food departments, including fresh meat and poultry, dairy, dry and packaged foods, and frozen foods) was converted into a Geographic Information System (GIS) usable format, by geocoding street addresses into store-point locations. Population data reported at the block level from the Census were aerially allocated down to 1/2-kilometer-square grids across the United States, where the distance was calculated from its geographic center to the center of the grid cell with the nearest supermarket. After linking this content with a city of Dallas shapefile, a map of the USDA-defined food deserts was created at the census tract level for Dallas, Texas.

### Food Deserts Mapping Using Yelp Data

Yelp is a commercial social media site founded in 2004 where individuals voluntarily write reviews of local businesses. Its users are able to give business ratings based on personal experience and an aggregation of the business' pricing, measured by the number of dollar signs allocated, ranging from 1 to 4. Since the Yelp Academic Datasets produced and released for use by the academic community were not available for Dallas, TX, the data were drawn by using the GraphQL query language for the Yelp Application Programming Interface (API) in 2018. The Yelp API data were queried to retrieve businesses categorized as a grocery store in the city. Multiple queries were run to include each zip code within the city to collect all possible grocery stores. To identify grocery stores labeled as a different category, six Yelp business categories (grocery stores, ethnic grocery stores, ethnic food markets, organic stores, health markets, and wholesale stores) were included in the query (25). Each business result provided a comprehensive list of business data, such as address, price, reviews, rating, and categories that identify the business. A specific query was run to limit the response content to only the business data relevant to this study. All query responses of grocery store data—name, address, and price rating—returned in JavaScript Object Notation (JSON) format were converted to comma-separated values (csv) format, checked for duplicates, and imported into ArcGIS Pro for georeferencing.

Geocoding led to the identification of 180 addresses associated with grocery stores within the city boundary. A 1-mile buffer was calculated for the grocery stores and the resulting file was used to clip the census tract shapefile. A Five-Year Estimates dataset (2012–2016) was downloaded from the American Fact Finder (the American Community Survey's Demographic and Housing Estimates, and Poverty Status in the Past 12 Months) and linked to the census tract boundary shapefile of the city of Dallas, downloaded from the Census Bureau's Cartographic Boundary Files website. Once the linked data were spatially joined with Yelp data, low access census tracts were identified based on areas spanning more than 1 mile away from a grocery store with an associated population ≥500 (following the USDA food deserts definition). This shapefile was joined with the American Community Survey's poverty data to get census tract food deserts and filtered to only include census tracts with at least 20% of the population living below the poverty level. The map of Yelp-based food deserts was then created at census tract levels for the city of Dallas and compared to the USDA-defined food deserts.

### Adjustment by Transit Score and Price Category

We then collected additional indicators to explore how healthy food accessibility would change and what new census tracts would be considered as potential food deserts, by incorporating additional factors affecting social and financial accessibility, such as public transportation and food price. The Transit Score data were drawn from the Walk Score™ website and used to measure the ability of the population that does not own automobiles to travel to nearby grocery stores using public transit. The raw Transit Score was calculated by taking the sum of the value of all nearby transit routes. Routes had a value based on the service level (frequency per week) multiplied by the mode weight (heavy/light rail weighted by 2, ferry/cable car/other by 1.5, and bus by 1) and a distance penalty. The distance penalty was estimated by a distance decay function using the distance to the nearest stop on a route. The raw Transit score was then normalized to a score between 0 and 10, and reported to the Walk Score website at the neighborhood level. The standardized transit scores for all 113 Dallas neighborhoods were converted to the census-tract level. The low-income census tracts that may have transportation barriers due to low transit scores were identified. Likewise, to assess how price categories for grocery stores from Yelp data may create additional barriers to healthy food access beyond income, all grocery stores in the data were classified by the price category provided by Yelp. Grocery stores with a high price category were used as an indicator of a location that may limit access to nearby residents due to price. The low-income census tracts that may present additional financial barriers due to high food prices were also identified and displayed over a map.

## Results

### Food Deserts Maps: USDA vs. Yelp Data

**Figure 1:**
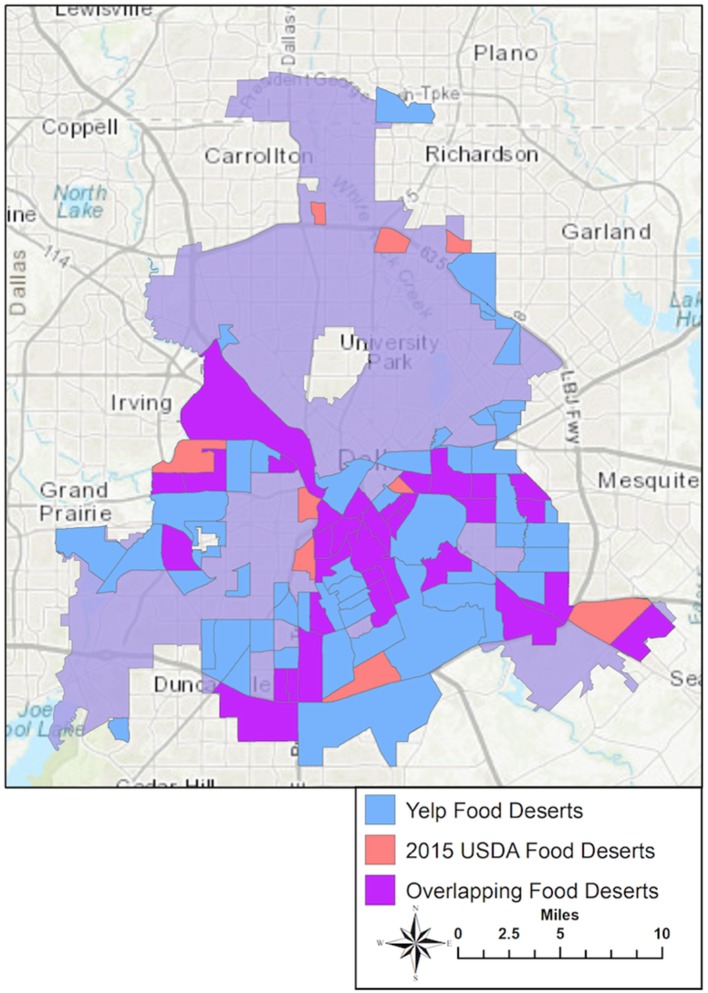
Census tracts designated as food deserts: USDA vs. Yelp data.

Both the 2015 USDA and Yelp data were used to draw food desert maps for the city of Dallas following the USDA's Food Access Research Atlas criteria, which were compared on the same map. Figure 1 shows that, among a total of 296 census tracts in the city, 9 census tracts were designated as food deserts based on the 2015 USDA data only (orange areas), 50 were identified as food deserts by the Yelp data only (blue areas), and 33 census tracts were overlapped by both data sources (purple areas). While 50 census tracts were newly identified as food deserts by Yelp data mostly in the southern area of Dallas, nine census tracts discovered from the USDA were not considered food deserts based on the Yelp data, most of which were located in vicinity areas. The census tracts that were newly designated as food deserts according to Yelp data could have lost healthy food accessibility during the time gap of the two data sources (2015–2018), but could have resulted from the different processes of constructing grocery store databases. Although both USDA and Yelp food desert maps were created based on the same criteria, they cannot be directly compared to one another due to the discrepancy in time of data collection (2015 vs. 2018). Despite the lack of comparability and capability to evaluate the accuracy of Yelp data, this study revealed a need of frequent update of the USDA's food desert designation using up-to-date source of information on grocery stores. The USDA data are supposed to be more exhaustive at the time of data collection, but the data may be limited in reflecting dynamic changes in grocery store presence over time. Our comparison demonstrated the feasibility of Yelp data as a free and real-time source of mapping for healthy food accessibility, not as a substitute but as a complement of the USDA data.

### Addition by Low Transit Score or High Price: Potential Areas of Concerns

**Figure 2:**
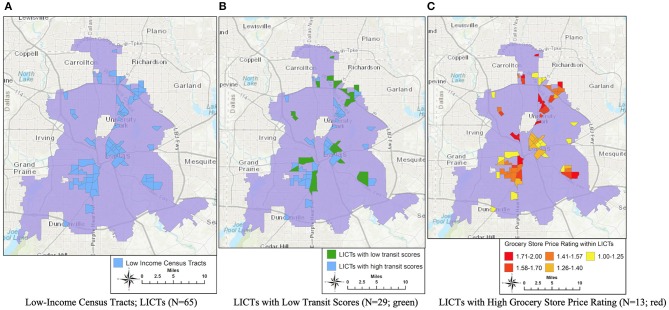
Low-Income census tracts with transit scores or grocery store price rating. **(A)** Low-Income census tracts LICTs (*N* = 65). **(B)** LICTs with low transit scores (*N* = 29; green). **(C)** LICTs with high grocery store price rating (*N* = 13; red).

Mapping based on crowdsourced data is not only dynamic (almost real-time), but also extendable to other purposes when combined with other variables from various sources of public or crowdsourced data. First, Figure 2A shows that there were 65 low-income census tracts (LICTs) in the city of Dallas, according to the USDA classifications. As visualized in Figure 2B, out of 65 LICTs, 29 census tracts had a transit score on the lower end of the scale (between 25 and 49; colored as green), meaning fewer nearby public transportation options exist in the community. None of the LICTs had a minimal transit score (between 0 and 24). This map shows that ~45% of LICTs may have households who face a lack of physical accessibility to healthy food if they live without a personal vehicle.

To explore if price data may reveal financial barriers in accessing healthy food in grocery stores, LICTs with at least one store within 1-mile were identified and joined with price descriptor data. As shown in Figure 2C, 13 LICTs were associated with the highest grocery store price rating (1.71–2.0; colored as red), which were scattered throughout low-income areas. Low-income households within these communities would still face limited accessibility to healthy food, despite living near grocery stores, as they cannot afford to purchase the food due to high prices.

These results highlight the point that distance, vehicle ownership and income are not the only factor that should be considered when assessing accessibility. Census tracts with low transit scores might not be considered as food deserts based on current metrics, but revealed other potential barriers to access healthy food. The same is true for the areas with high-price grocery stores nearby. This study raised the point that the USDA's conventional designation of food deserts may need to be reconsidered and refined with a more sophisticated conceptualization and methodology by including more relevant and readily available variables.

## Discussion

This study echoed previous studies using Yelp data as a source to identify businesses as commercially available data sources, but also revealed the limitation due to its incomplete coverage. We found that more census tracts were identified as food deserts using Yelp data compared to the USDA data, possibly because of the declining trend of healthy food accessibility in the southern part of Dallas, Texas. However, another likely reason may be that a good number of grocery stores were not listed as businesses in Yelp due to the absence of user-generated content. The findings of this study cannot confirm which dataset (USDA vs. Yelp) matches the real world more accurately due to the time difference between the two data sources, but could show the potential of the crowdsourced georeferenced data which are free and readily available for routine mapping. In this regard, Yelp data may be used to track ongoing efforts of addressing healthy food access with more frequency when a large-scale primary data collection is not possible. To sum up, Yelp data is still incomplete in coverage and limited for wide application, but has potential to be improved in the near future as crowdsourcing technology and platforms rapidly evolve.

We also extended the exploration of Yelp data's utility in food access research, by integrating price descriptors with geocoded grocery stores, which shows the possibility of locating census tracts in cities where grocery prices may limit access to healthy foods. This study also illustrated that a variety of publicly available records, such as public transit or walkability score, may be used as additional layers for public health mapping. Given the sizable proportion of low-income census tracts with low transit and high price rating, further investigation is warranted to explain transportation and financial barriers in healthy food access in these communities. However, due to the data issue, we failed to create a map explicitly showing the areas newly designated as food deserts by additional factors, which should be performed in the future study. Moreover, future research could identify what price is actually being measured through targeted qualitative work.

This study does not aim to evaluate the accuracy of the Yelp data nor suggest it to replace the existing USDA food desert mapping. Rather, it seeks to provide more evidence to demonstrate the role of crowdsourced data in comprehensive and timely mapping of healthy food accessibility. The resulting food desert maps would be more comprehensive and reliable when the USDA and Yelp lists of grocery stores collected at the same time are systematically combined. Other limitations of this study include the lack of validation of Yelp data and incomplete information on actual food costs in identified stores. This study shed light on the need for an on-the-ground direct observation of food inventory to better interpret the data presented in this study, because the goods offered vary greatly between national chain grocery stores and small food retailers (8). To address this issue of healthy food access at the local level, place-specific strategies have been suggested that rely more on local area sources in describing healthy food access (10, 27). Future work should include multiple cities across the US to demonstrate the representative quality of Yelp data across the country. Moreover, the method used in this study could give guidance to future studies, not only on food deserts but also on other public health mapping such as noise mapping (28) and pollution mapping (29).

## Data Availability Statement

The datasets generated for this study are available on request to the corresponding author.

## Author Contributions

TM and DK designed the study. TM performed GIS database construction and mapping. DK and SS interpreted the results. DK and TM wrote and revised the manuscript in consultation with SS. All authors discussed the results and contributed to the final manuscript.

### Conflict of Interest

The authors declare that the research was conducted in the absence of any commercial or financial relationships that could be construed as a potential conflict of interest.
